# Projected changes in bird assemblages due to climate change in a Canadian system of protected areas

**DOI:** 10.1371/journal.pone.0262116

**Published:** 2022-01-21

**Authors:** Marcel A. Gahbauer, Scott R. Parker, Joanna X. Wu, Cavan Harpur, Brooke L. Bateman, Darroch M. Whitaker, Douglas P. Tate, Lotem Taylor, Denis Lepage

**Affiliations:** 1 Canadian Wildlife Service, Environment and Climate Change Canada, Ottawa, Ontario, Canada; 2 Parks Canada, Tobermory, Ontario, Canada; 3 National Audubon Society, New York City, New York, United States of America; 4 Parks Canada, Rocky Harbour, Newfoundland and Labrador, Canada; 5 Parks Canada, Nipigon, Ontario, Canada; 6 Birds Canada, Port Rowan, Ontario, Canada; USDA Forest Service, UNITED STATES

## Abstract

National parks often serve as a cornerstone for a country’s species and ecosystem conservation efforts. However, despite the protection these sites afford, climate change is expected to drive a substantial change in their bird assemblages. We used species distribution models to predict the change in environmental suitability (i.e., how well environmental conditions explain the presence of a species) of 49 Canadian national parks during summer and winter for 434 bird species under a 2°C warming scenario, anticipated to occur in Canada around the mid-21st century. We compared these to existing species distributions in the 2010s, and classified suitability projections for each species at each park as potential extirpation, worsening, stable, improving, or potential colonisation. Across all parks, and both seasons, 70% of the projections indicate change, including a 25% turnover in summer assemblages and 30% turnover in winter assemblages. The majority of parks are projected to have increases in species richness and functional traits in winter, compared to a mix of increases and decreases in both in summer. However, some changes are expected to vary by region, such as Arctic region parks being likely to experience the most potential colonisation, while some of the Mixedwood Plains and Atlantic Maritime region parks may experience the greatest turnover and potential extirpation in summer if management actions are not taken to mitigate some of these losses. Although uncertainty exists around the precise rate and impacts of climate change, our results indicate that conservation practices that assume stationarity of environmental conditions will become untenable. We propose general guidance to help managers adapt their conservation actions to consider the potentially substantive changes in bird assemblages that are projected, including managing for persistence and change.

## Introduction

The on-going and projected impacts of climate change to the survival and distribution of biodiversity is global in scale [1–3] and is even felt within protected areas that have been established to safeguard and conserve species [4–8]. Climate change creates an increasingly complex and uncertain context for protected areas, one which may render unachievable traditional conservation goals, such as maintaining current or historic assemblages of species [9–12]. Indeed, because protected areas are often established in extreme or atypical landscapes (e.g., high latitudes, high elevations, coastal areas, or arid regions) they may be disproportionately affected by climate change [6]. However, the challenges associated with biodiversity conservation would be even greater without protected areas [13–15], and managers of such sites must therefore have access to information on how to adapt effectively in this period of rapid environmental change.

Since 1948, when nation-wide records have been available, Canada has experienced an increase in mean annual temperature and a general increase in total annual precipitation, and these changes are expected to continue or even accelerate because of climate change [16]. These changes have triggered habitat alteration through shifts in factors such as snow and ice cover, wildfire regimes, sea level, hydrology, vegetation composition and structure, and growing season phenology [17–19], all of which influence the distribution and extent of suitable environmental conditions for species. While each species has a preferred range of climatic suitability, responses to changing conditions may vary [20–23]. Some species may be unaffected or find a means to adapt to local conditions, such as by advancing the timing of nesting [24], while others are more sensitive and less adaptable and will need to either shift their range or face extirpation or extinction [25, 26].

There has already been a substantial decline in North American bird populations over the past half-century, with concern that ongoing and future threats will result in further losses [27]. To examine climate-induced changes in bird populations, near-future suitability projections can be used [28], and have already been shown to estimate contemporary species distribution shifts [29]. Projecting potential changes in bird assemblages in an area can reduce future uncertainty and inform more effective conservation actions.

As climatic conditions change and bird assemblages are modified, there will also be shifts in functional traits [30, 31] of the assemblage that reflect the ecological roles of species (e.g., seed dispersal, biological control, pollination), as well as the processes (e.g., nutrient cycling, energy flow, productivity) that characterize and maintain an ecosystem [32–34]. As assemblages change, taxon-free indicators such as functional traits will become increasingly useful in conservation efforts, including monitoring and assessing ecosystem state [12].

Our primary objective was to describe projected changes in environmental suitability for bird assemblages (i.e., species composition) in Canada’s national park system, similar to work previously completed for the U.S. National Park Service [35]. Additionally, we updated and used 2019 distribution models and examined the functional traits of the current and future assemblages, to provide some understanding of potential changes to ecosystem function. By classifying parks according to relative trends in species turnover, we also begin to identify possible adaptation options and pathways for consideration by managers. We expect these projections to also help inform the public and partners about the potential impacts of climate change in Canada more broadly, and provide guidance for conservation and stewardship activities [30].

## Methods

### Study area

We based our study on the 2018 system of 50 Canadian national parks, national marine conservation areas, and national urban park, spanning all provinces and territories (S1 Fig); hereafter “parks”. Although there are differences in designations, they are all administered by Parks Canada to protect nationally significant examples of Canada’s natural and cultural heritage and to represent the country’s terrestrial and marine natural regions [36, 37]. We excluded only Quttinirpaaq from analyses, as it was beyond the northern limits of the developed models, leading to a final set of 49 parks, encompassing 297,844 km^2^ of land and 10,089 km^2^ of marine and fresh waters, with an average park size of 6,284 km^2^ (median = 1,377 km^2^, range 13 km^2^ to 45,554 km^2^). For the national marine conservation areas, we limited analyses to the islands and coastal components. We classified parks into the ecological regions defined in Marshall et al. 1999 [38].

### Data and species distribution modelling

We used previously published species distribution model (SDM) projections for 604 North American bird species [39, 40] to project change in environmental suitability as a value from 0–1, with a score of 1 indicating environmental conditions that best explain the presence of a species. The underlying models incorporated over 58 million bird occurrence records from 70+ data sources, such as eBird, North American Breeding Bird Survey, Global Biodiversity Information Facility, and NatureCounts (see Bateman et al. 2020 [39] for list of data sources). They accounted for 27 bioclimatic variables, vegetation types, terrain ruggedness, and anthropogenic land cover, as well as water presence for relevant birds. Models related occurrence with environmental data and were built using boosted regression trees (BRTs) and maximum entropy (MaxEnt), which are appropriate for exploring non-linear species-habitat relationships [41, 42]. The best performing model (i.e., baseline models for 2010s) was selected for each species and season using median AUC. These baseline models were then used to project future suitability at +2.0°C increase in global mean surface temperature above pre-industrial levels, which is anticipated to occur around mid-century (2041–2070) under the RCP8.5 (Representative Concentration Pathway) scenario [43, 44]. SDMs used an ensemble of 15 general circulation models (GCMs) that capture a range of bioclimatic conditions, and a model consensus approach to assess agreement across GCMs, calculated multiple model thresholds (including mean occurrence, maximized sensitivity + specificity, maximum Kappa, and others), and utilized expert opinion combined with a minimal decrease in model performance to select a final species-specific threshold above which a species is likely to occur (see Bateman et al. 2020 [39] for details). The result was 1x1 km resolution surfaces for each bird species and season with suitability values based on the percent change between the +2.0°C scenario compared to a 2010s baseline suitability. Bateman et al. 2020 [39] classified each 1x1 km cell as: 1) Potential extirpation: species was present in 2010 models but not under the +2.0°C scenario; 2) Worsening: -100% to -25% change in suitability; 3) Stable: -25% to 25% change; 4) Improving: >25% change in suitability; 5) Potential colonisation: species was not present in 2010s models but suitability improves to above the species-specific threshold. For this study, we rasterized park boundaries to match the 1x1 km cell resolution of the models. We summarized suitability change for each species and season across all cells each park comprises. For each species, we assigned summer and winter projections for a park based on which of the five categories above is associated with the largest number of cells within each park. We considered species with extirpation, worsening, stable, and improving projections as present in the 2010s. We considered species with worsening, stable, improving, and potential colonization projections as present under the +2.0°C scenario. We conducted these analyses in R versions 3.4.4 and 4.0.2 using *raster* and *tidyverse* packages.

### Species assemblages

Because climate-based species distribution models project locations of suitable conditions rather than definitive occurrence [45], and our aim is to provide park managers with the most realistic results possible, we systematically reviewed species outputs. We compared the species modelled as currently being present against contemporary lists for each park, based primarily on the NatureCounts database [46], which compiles data from a wide variety of citizen science programs and avian monitoring efforts. For each of summer and winter, species lists from modelled projections were reviewed by Parks Canada staff and other regional experts, and records considered to be from transient, migrant, or accidental species were excluded from the 2010s baseline projection to avoid overprediction. We removed 17% of modelled records from the analyses because their presence was unconfirmed.

To increase model accuracy, we also filtered out future model projections that represented a highly improbable colonisation scenario based on distance or habitat. Examples include projections of isolated records >1000 km beyond projected future range limits, species crossing the continent from the Pacific to eastern Arctic or Atlantic coasts or from the Rocky Mountains to Labrador without intermediate records, prairie species projected to colonise mountain parks, marine species projected to colonise inland waters, and conifer-crop dependent species projected to colonise high Arctic parks where trees may start to establish in the coming decades but would not grow sufficiently by 2041–2070 to support these birds. We removed 3.2% of records for which the projected future trend was considered as being improbable.

With the final set of present (2010s) and future (+2.0°C scenario) species, we calculated metrics comparing the present and potential future species assemblages within each park under a +2.0°C warming scenario. We chose the Sørenson similarity index [47] to quantify species turnover as it is based on presence-absence data, and compared to the Jaccard index, has a slightly higher focus on shared species than outliers [48]. We also measured species richness (SpRich), the raw number of species present at a park [49].

To understand how parks compare to each other, we classified them into relative groups based on the proportion of potential colonisations to potential extirpations, following Hole et al. 2011[50]. We plotted the proportion of colonisations against the proportion of extirpations, then divided the resulting plot into five sectors based on the median and quartiles: high turnover, high potential extirpation, high potential colonisation, intermediate change, and low change. We calculated Sørenson similarity index using the vegdist function in the R (version 3.4.4) package *vegan*.

### Species functional indices

Preliminary analysis of functional traits indicated extreme outliers in the Arctic Region parks and Sable Island. We therefore excluded these nine parks from the functional analyses to avoid improperly skewing the overall results.

To quantify changes in functional traits, we created a species-by-trait matrix for the modeled bird species. We classified each species with respect to five functional traits based on Rodewald 2015 [51]: 1) feeding behavior; 2) primary food type; 3) preferred habitat; 4) nesting behavior; and, 5) relative size. These categories describe resource needs (e.g., type and amount) and the behaviors employed to obtain them. We used the daisy function of the *Cluster* package version 2.1 in R (version 4.0.4) [52] to convert the species-by-trait matrix into a distance matrix using Gower distance, which is commonly used to represent functional distances that are characterized by both quantitative and qualitative variables. We used a principal coordinates analysis to create the functional space representing the distance matrix. We used six axes to calculate the functional metrics, as this enabled functional richness to be calculated for all parks, including those with lower species richness [53, 54], it avoided computational problems derived from high-dimensional convex hulls (e.g., 10 dimensions), and it provided an improved fit over lower-dimensional spaces (mean squared deviation summer = 0.021, winter = 0.017, assessed using the quality_funct_space function; [55]).

Functional richness quantifies the proportion of trait space occupied by a given assemblage, by comparing a convex hull linking the species with the most extreme trait values within an assemblage to the volume occupied by the maximum convex hull that can be created using the global species pool [53]. Functional dispersion represents the functional variability within an assemblage by measuring the mean dispersion of an assemblage’s species from its centroid in trait space [54]. For comparison across parks and between current and projected assemblages, we scaled dispersion values to the maximum dispersion value possible, given the global species pool (i.e., an assemblage with only the two most distant species). Occurrence data can be used to describe both the range of functional space an assemblage occupies (functional richness) and diversity within an assemblage (functional dispersion) [54, 56]. Higher levels of either are viewed as an assemblage being more buffered against environmental perturbations (i.e., greater capacity to respond to change) [30, 54, 57]. We calculated functional richness and dispersion using the dbFD function in the *FD* package in R (version 4.0.4) [54, 58].

We used the taxon restrictedness function in the *funrar* package (version 1.2.1) in R (version 4.0,4) [59]. Taxon restrictedness is a regional-scale index that represents a species distribution across parks scaled between zero and one. For this study a taxon restrictedness value of zero indicates a species is absent from all parks (i.e., completely restricted) and a value of one indicates a species is present at all parks (i.e., no restriction) [60, 61]. For this analysis we analyzed only species that occurred in both current and future communities in each season (356 species in summer; 215 species in winter).

We assessed functional change between current and projected assemblages for each of the above indices, plus species richness, using model II major axis (MA) regression, with the lmodel2 function in the *lmodel2* package version 1.7.3 in R (version 4.0.4) [62]. We chose model II MA regression because both the x and y variables are in the same units, subject to error, and we did not assume a causal relationship between them [63, 64]. We assessed the significance of the model parameters and the correlation coefficient using 999 permutations to determine whether the communities differed significantly from the null model of a 1:1 relationship.

## Results

**Fig 1:**
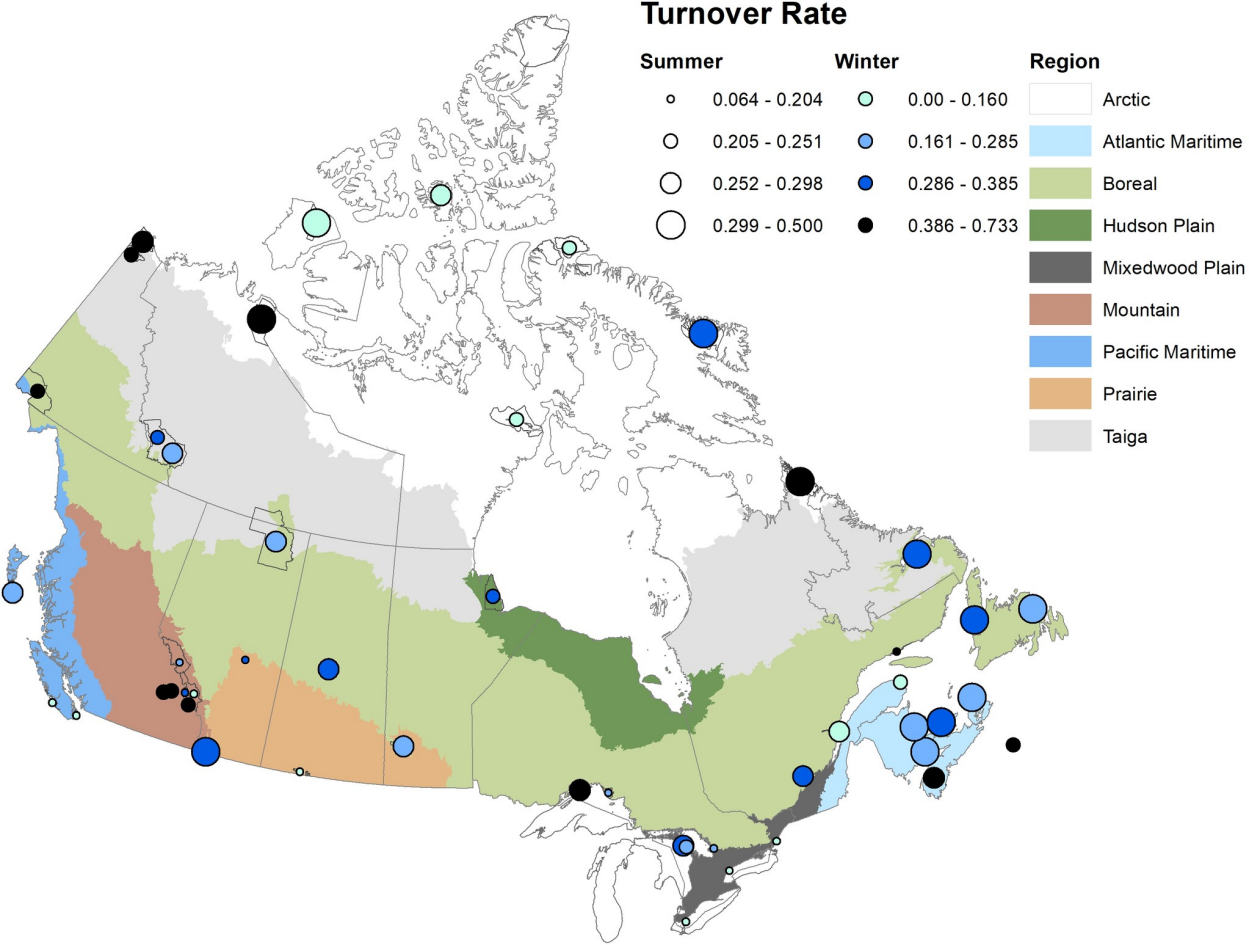
Projected species turnover (Sørenson) for +2.0°C warming scenario across all 49 parks, with 0 being no change and 1 being complete. Values can also be understood in terms of percent turnover (e.g., 0.252 = 25.2%). Circle diameter represents summer rates and color represents winter rates. Breaks in classes are based on quartiles.

**Fig 2:**
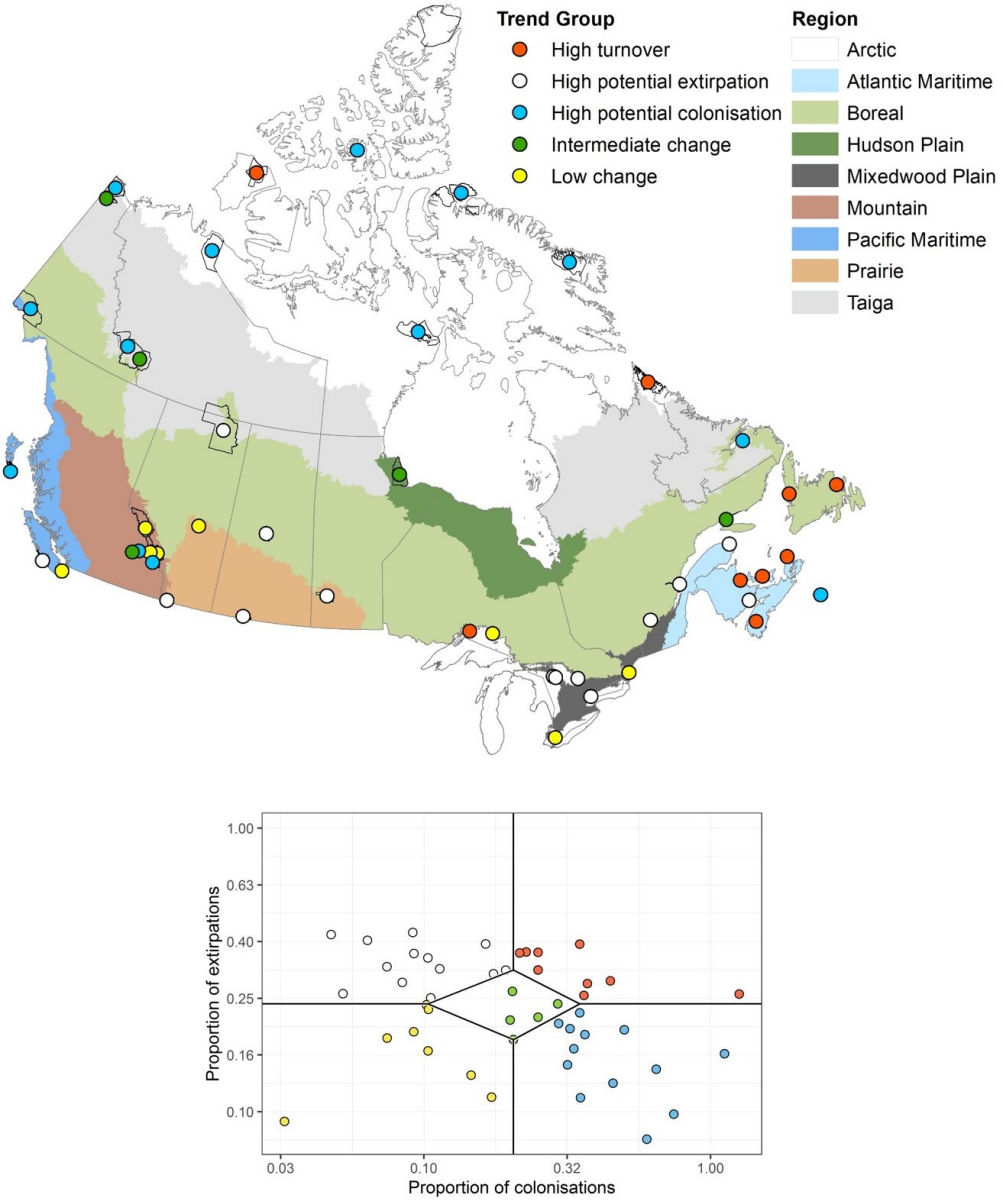
Classification of parks into relative trend groups based on the proportion of potential colonisations to potential extirpations in summer. Each circle represents a park and its modeled projection.

The final analyses of 49 parks included 434 species (424 summer and 272 winter). The average number of species analyzed per park for summer was 137.2 ± 6.2 (SE) (range 16–201) and for winter was 70.0 ± 5.6 (range 1–159). A complete list of species and trends can be found in S1 Table. By mid-century, the average projected species turnover per park is 24.9% in summer and 30.3% in winter, as measured by the Sørenson similarity index (Tables 1 and S2). The greatest projected rates of summer turnover are in two of the high Arctic region parks, Auyuittuq and Aulavik, at 42% and 33%, respectively (S2 Table), but species turnover rates are also projected to be high among many of the parks along Canada’s east coast, including some in both the Atlantic Maritime and Boreal regions (Fig 1). Conversely, over half of the parks projected to have low turnover are in the Mountain and Mixedwood Plain regions (Fig 2).

**Table 1:** Regional and national summaries of potential changes in bird species with +2.0°C warming: 1) # of parks used in regional and national calculations; 2) mean (± SE) change in species richness per park; 3) mean (± SE) number of potential colonisation per park; 4) mean (± SE) number of potential extirpations per park; and, 5) mean (± SE) change in Sørenson similarity turnover index per park.

Region	# of Parks	Species Richness		Potential Colonisation		Potential Extirpation		Turnover	
		Summer	Winter	Summer	Winter	Summer	Winter	Summer	Winter
Arctic	7	19.6 ± 6.5	7.1 ± 5.2	26.7 ± 7.0	7.1 ± 5.2	7.1 ± 1.5	0	0.32 ± 0.04	0.24 ± 0.10
Atlantic Maritime	7	-12.6 ± 5.7	29.3 ± 6.1	21.9 ± 4.0	35.4 ± 5.3	34.4 ± 6.1	6.1 ± 1.5	0.29 ± 0.02	0.32 ± 0.07
Boreal	12	-18.4 ± 9.0	23.1 ± 3.7	24.8 ± 4.3	29.1 ± 3.4	43.3 ± 5.7	6 ± 0.9	0.26 ± 0.02	0.32 ± 0.03
Hudson Plain	1	4	15	29	15	25	0	0.23	0.31
Mixedwood Plain	6	-20.5 ± 3.7	20.8 ± 6.0	12.5 ± 2.2	26.3 ± 5.8	33 ± 3.5	5.5 ± 0.7	0.19 ± 0.02	0.19 ± 0.04
Mountain	7	-3.3 ± 10.6	33.6 ± 6.4	24.7 ± 4.6	38.1 ± 6.0	28 ± 8.1	4.6 ± 1.3	0.19 ± 0.03	0.44 ± 0.09
Pacific Maritime	3	-3 ± 13.4	30.3 ± 1.5	19.3 ± 8.4	34.7 ± 1.5	22.3 ± 6.0	4.3 ± 0.3	0.21 ± 0.05	0.16 ± 0.02
Prairie	1	-24	13	10	13	34	0	0.2	0.16
Taiga	5	16.4 ± 7.4	19.8 ± 4.6	38.6 ± 5.9	19.8 ± 4.6	22.2 ± 3.9	0	0.26 ± 0.02	0.38 ± 0.05
**National**	49	-5.4 ± 3.7	22.7 ± 2.2	24 ± 2.0	26.6 ± 2.2	29.4 ± 2.6	3.9 ± 0.5	0.25 ± 0.01	0.30 ± 0.03

Across all species, parks, and both seasons, 70% of projections of suitability (i.e., how well environmental conditions explain the presence of a species) are expected to change (i.e., improving or worsening suitability, or potential colonisation or extirpation, S1 Table). For parks where one or more species are projected to experience potential colonisation or potential extirpation (i.e., all 49 parks in summer, 46 parks in winter), potential extirpation exceeds potential colonisation in 55% of parks in summer, whereas in winter potential colonisation exceeds potential extirpation everywhere except Rouge National Urban Park, where the rates are projected to be equal. Overall, the number of potential coloniser species is much higher in winter than in summer (Table 1).

**Fig 3:**
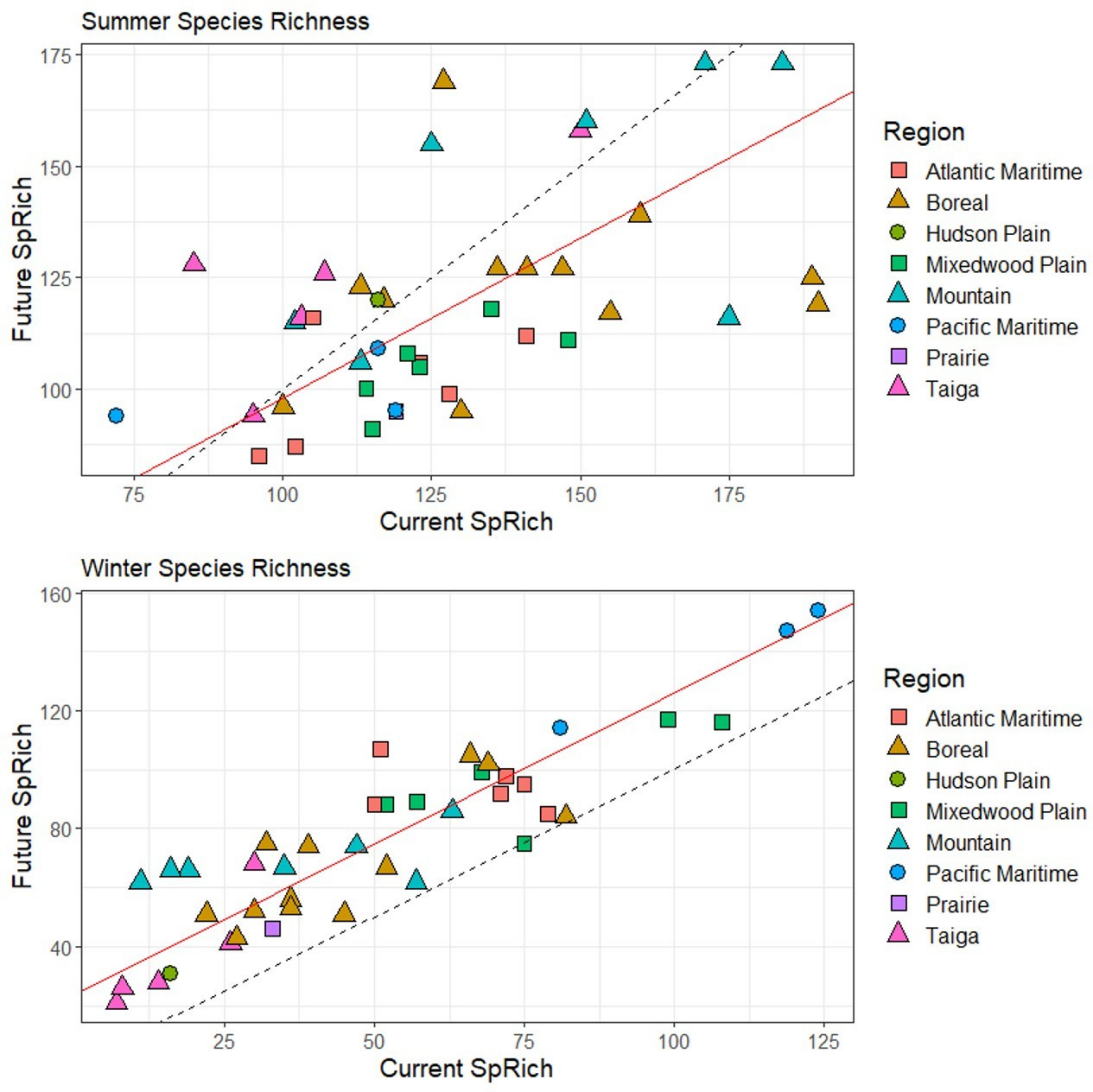
Change between current and future species richness (SpRich) across 41 parks (classified by region) for both summer and winter bird assemblages. The dashed line represents the null model (1:1 relationship); the red line is the model II major axis regression line (summer y = 26.38 + 0.72x, r = 0.53; winter y = 23.84 + 1.02x, r = 0.89).

**Fig 4:**
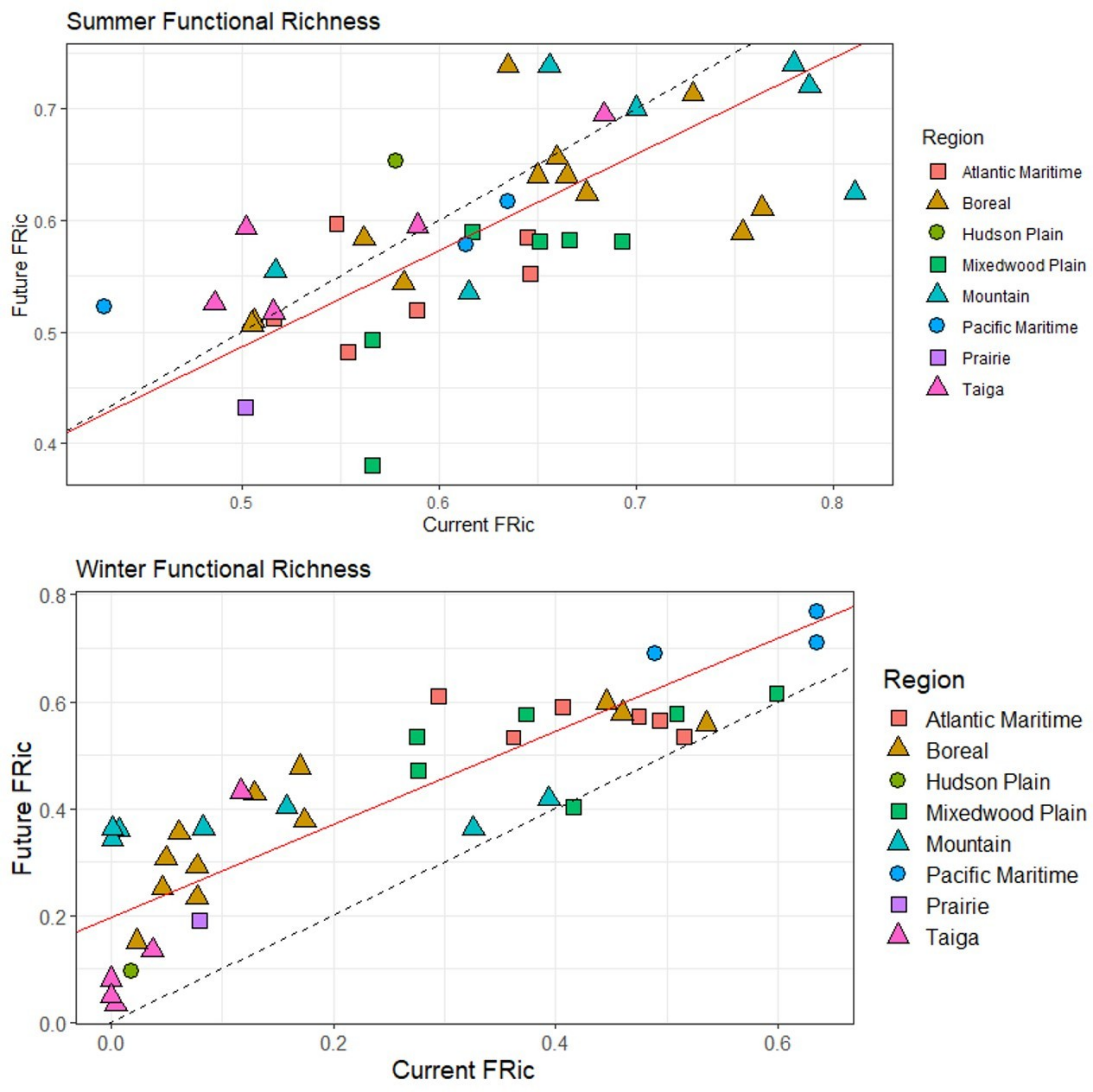
Change between current and future functional species richness (FRic) across 41 parks (classified by region) for both summer and winter bird assemblages. The dashed line represents the null model (1:1 relationship); the red line is the model II major axis regression line (summer y = 0.06 + 0.86x, r = 0.66; winter y = 0.20 + 0.87x, r = 0.86). Functional richness represents the volume occupied by a park in multidimensional trait space.

**Fig 5:**
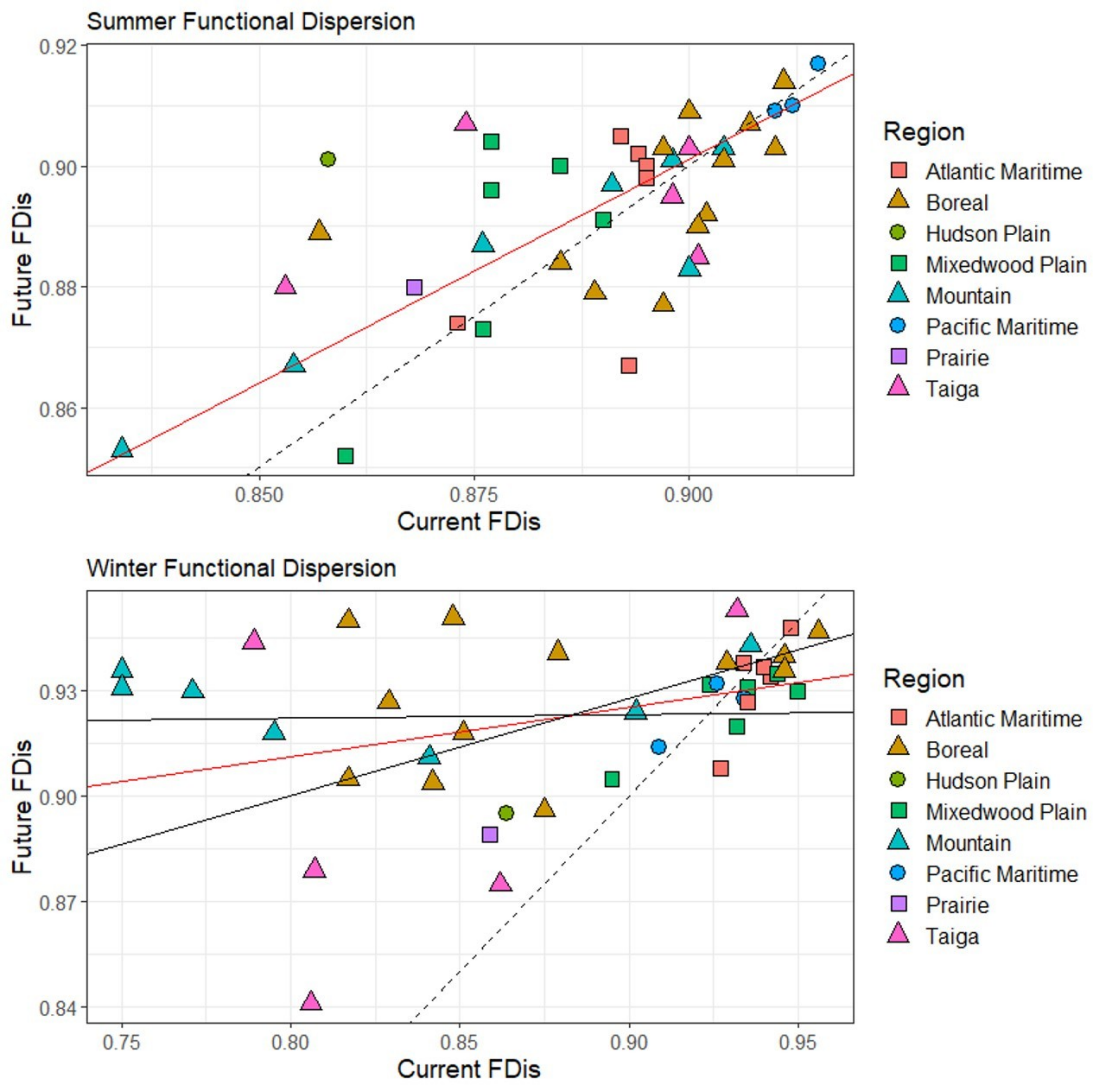
Change between current and future functional dispersion (FDis) across all 41 parks (classified by region) for both summer and winter bird assemblages. The dashed line represents the null model (1:1 relationship); the red line is the model II major axis regression line (summer y = 0.23 + 0.74x, r = 0.66; winter y = 0.80 + 0.14x, r = 0.33). Functional dispersion represents the mean distance of all species to the centroid of the assemblage in multidimensional trait space.

**Fig 6:**
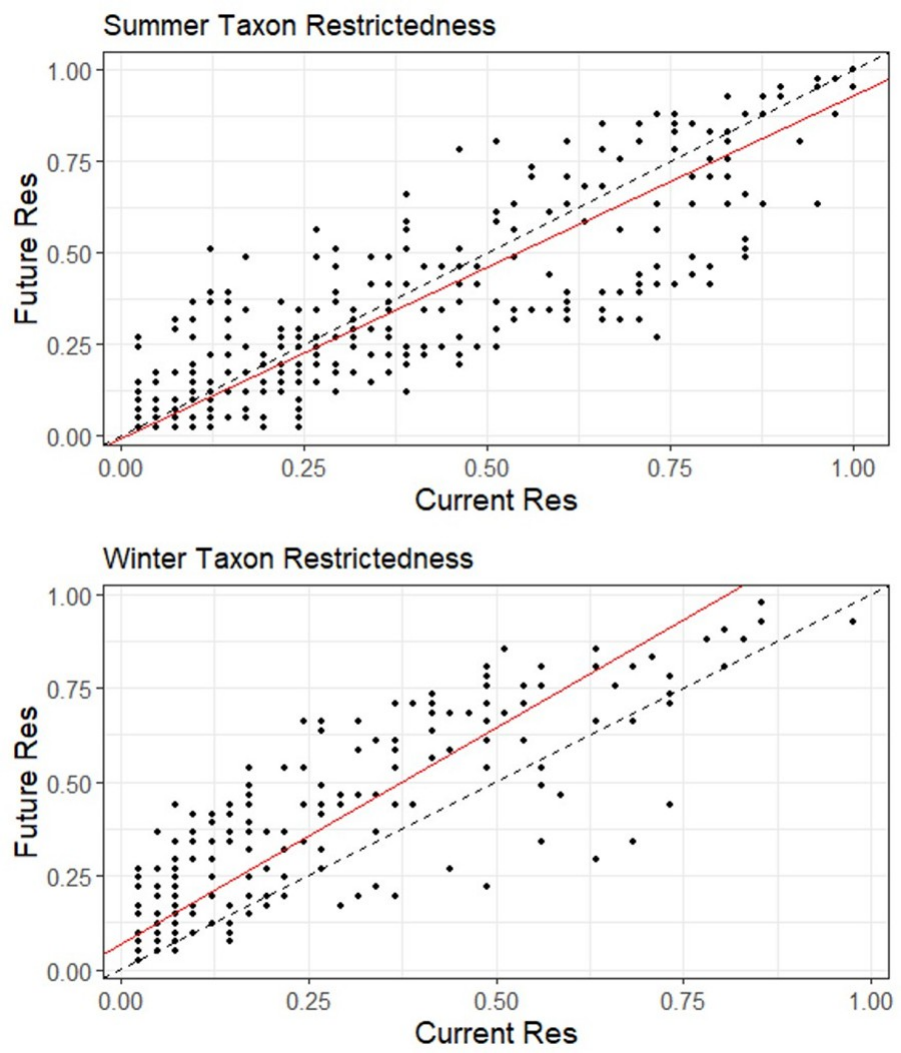
Change between current and future taxon restrictedness (TRes) across all 41 parks (classified by region) for both summer and winter bird assemblages. The dashed line represents the null model (1:1 relationship); the red line is the model II major axis regression line (summer y = (-0.01) + 0.94x, r = 0.86; winter y = 0.07 + 1.15x, r = 0.85). Restrictedness represents an index value between 0 and 1, where 0 indicates a species is absent from all parks (i.e., completely restricted) and 1 indicates present at all parks (not restricted).

Figs 3–6 (S3 and S4 Tables) illustrate the changes between current and future species assemblages in terms of species richness, functional richness, functional dispersion, and taxon restrictedness. In each case, Model II MA regression analyses found a significant positive correlation between the current and projected future conditions. However, correlation was stronger in winter for species richness and functional richness, and stronger in summer for functional dispersion (Table 2).

**Table 2:** Slope and intercept values, including 95% confidence intervals (C.I.), and regression coefficients for the Model II MA Regression models comparing the species richness (SpRich), functional richness (FRic), functional dispersion (FDis), and taxon restrictedness (TRes) between current and projected bird assemblages (null model 1:1).

Index	Season	Slope (C.I.)	Intercept (C.I.)	R
SpRich	Summer	0.72 (0.40–1.18)	26.38 (-33.47–67.74)	0.53*
	Winter	1.02 (0.87–1.20)	23.84 (14.60–31.67)*	0.89*
Fric	Summer	0.86 (058–1.24)	0.06 (-0.18–0.22)	0.66*
	Winter	0.87 (0.72–1.06)	0.20 (0.15–0.24)*	0.86*
FDis	Summer	0.74 (0.50–1.07)	0.23 (-0.05–0.45)	0.66*
	Winter	0.14 (0.01–0.28)*	0.80 (0.67–0.91)*	0.33*
Tres	Summer (n = 356)	0.94 (0.88–0.99)*	-0.01 (-0.03–0.11)	0.86*
	Winter (n = 215)	1.15 (1.06–1.25)*	0.07 (0.04–0.089)*	0.85*

Values with “*” indicate statistical significance (p<0.05). Forty-one parks were used for the SpRich, FRic, and FDis analyses, whereas n indicates the number of bird species used in the TRes analysis.

In summer, no significant shifts are projected at a national scale for any of species richness, functional richness, and functional dispersion, indicating no systematic change between current and future assemblages. Rather, the analyses project a mix of increases and decreases among parks. Summer species richness is projected to increase in most Taiga and Mountain parks, and to decline in most Atlantic Maritime and all Mixedwood Plains parks (Fig 3); the pattern is similar for functional richness, other than there are both increases and decreases among the Mountain parks (Fig 4). Projections of functional dispersion are more varied, with no regional patterns apparent (Fig 5). Overall taxon restrictedness is expected to change little in summer, although the distribution of common species will be reduced more than rare species (Fig 6).

Greater differences are projected in winter. Species richness is expected to increase in all regions, most notably in the Mountain and Atlantic Maritime parks (Fig 3). Similarly, functional richness is projected to increase in all regions, though none stand out, and instead it is individual parks with the lowest current functional richness that on average are expected to see the greatest gains (Fig 4). Future functional dispersion is expected to vary much less among parks than it does currently, with most of the largest gains occurring among the Mountain and Boreal parks, and only minor declines in a small number of parks, mostly in the Mixedwood Plain and Atlantic Maritime regions (Fig 5). The model trend crossed the 1:1 line (null model of no change) showing that the parks with the highest current functional dispersion are projected to decrease; however, the low correlation value (r = 0.33) indicates there was high variability around the overall trend. In winter, taxonomic restrictedness models show that most species are projected to expand their distribution with common species expanding most (Fig 6).

## Discussion

By mid-century, climate change is expected to have substantial impacts on bird communities in Canada’s system of national parks, with a potential turnover (change in species composition) of 24.9% in summer and 30.3% in winter. Environmental suitability is projected to be maintained for only 30% of species occurrences across seasons and parks. However, our results suggest considerable variability in the distributional shift of individual species, and in the resulting changes in community composition at individual parks.

Overall, the number of potential coloniser species is expected to be much higher in winter than in summer, consistent with the finding that wintering bird communities in Europe and North America are more rapidly tracking suitable habitat than summer breeding communities [65]. This is partly attributable to many species that are likely to become year-round residents in regions of Canada in future, especially in southern parks but also to a lesser extent in the north (e.g., Snow Bunting, *Plectrophenax nivalis*, in Auyuittuq). This includes winter colonisation by some species considered to be broadly declining in Canada, such as Cassin’s Finch, *Haemorhous cassinii* and Red-headed Woodpecker, *Melanerpes erythrocephalus* [66, 67]. Future changes are difficult to predict with accuracy in complex, interrelated ecological systems, and climate change is likely to catalyze other stressors such as species invasion, habitat loss, water quality degradation [68], changes in species interactions [69], and many other factors that affect bird survival but cannot be readily modeled. However, there is evidence that some appear to already be expanding their range in Canada. For example, as our models projected, Red-bellied Woodpecker, *Melanerpes carolinus* and Northern Cardinal, *Cardinalis cardinalis* are steadily moving north in Ontario and into southern Quebec [70–72], while Black-necked Stilt, *Himantopus mexicanus* and White-faced Ibis, *Plegadis chihi*, are substantially increasing their presence in the Prairie region [72, 73]. Conversely, other species may expand their range more slowly than the shift in climate if they are less successful at gaining a share of already occupied habitats, such as White-eyed Vireo, *Vireo griseus* and Tufted Titmouse, *Baeolophus bicolor*, that have largely stalled their northward movement into Ontario since the early 2000s [72].

An important limitation of our results is that the underlying species distribution models do not definitively outline future ranges; rather, they should be interpreted as projecting a potential distribution based on changes in broad-scale factors such as climate and vegetation. Although climate is a dominant driver of bird distributions in North America [74], models developed using only climate variables can lead to overestimates of species’ distributions [75]. We therefore included additional factors such as land cover, habitat availability, and topography in the models, but recognize that actual changes in species distribution can also be influenced through park-based factors such as habitat quality, food abundance, and interspecific interactions. We attempted to account for this with park-based independent validation of contemporary occurrence data, but future projections could still overestimate range expansion and potential colonisation.

Also of note, several of the parks in our study are close to others, in some cases even sharing a common border (S1 Fig). There is some lack of independence among these parks, and correspondingly a degree of auto-correlation in their model output. However, examination of the models showed that contemporary assemblages and projected changes can differ considerably even in immediately neighbouring parks, based on factors such as topographic diversity and being just within or slightly beyond the limits of shifting species distributions. For example, although most of the Mountain region parks are closely clustered, projected colonisation rates vary substantially. Choosing to include certain parks but exclude others would require development of a prioritisation framework and would obscure some locally specific predictions with valuable management implications. We therefore retained all parks covered by the model, while acknowledging that the inclusion of adjacent parks may give some proportionately greater weight to the Mountain and Taiga regions in particular. Furthermore, any clear effect of park size may be masked by correlations between park size and factors such as latitude, remoteness, and adjacent land use, as parks in more developed southern regions are typically much smaller than those in remote northern locales. Despite these limitations, species distribution modelling does have utility for priority-setting and targeting species and habitat management [76], especially when it can be combined with local knowledge to more comprehensively assess species needs in a given area [77, 78].

Although bird communities in protected areas may be more resistant and resilient to climate change than those in more developed or working landscapes [15, 79, 80], protected areas remain vulnerable and there is already evidence of changes to terrestrial biomes and bird assemblages in some of Canada’s national parks [26, 81–84]. As our findings illustrate, substantial further changes are expected, and although they appear to be park- and species-specific, some regional patterns are evident. For example, the relatively low rates of change projected in most of the Mountain region parks are consistent with theories of climate refugia occurring in areas of high topographic diversity that allow for different climates to exist in close proximity, and perhaps persist, through variation in attributes such as solar exposure, snow persistence, air flow, water inputs and canopy cover [85]. Topographic variability thus increases the likelihood that similar climatic conditions to those being lost might continue to occur nearby and be within reach of volant species such as birds [86]. Habitat connectivity is also an important factor [87], and in the case of some parks in the Mountain region (e.g., Banff, Jasper, Yoho, Kootenay) the proximity of adjacent protected areas and associated habitat may help bird assemblages persist regionally.

In the Arctic region a very different pattern is emerging. All of the parks in this region are projected to experience high potential colonisation or high turnover (i.e., both high colonisation and high extinction rates; Fig 2). This is not surprising, as Arctic regions are among those experiencing the most rapid change globally [2]. These parks are very different from those in the Mountain region noted above, as they are lacking in canopy cover and (except for Auyuittuq and Torngat Mountains) are largely flat, and therefore have little capacity for topographically supported climate refugia. On the contrary, conditions in the Arctic region parks can promote positive feedback loops with negative climatic consequences, such as decreased ice cover and increased thawing of permafrost and associated carbon emissions [88]. Changes in vegetation, and resulting habitat for bird species, are already being seen in the Arctic region [84, 89–91]. However, our models show a limit to northward range shifts, consistent with other studies [92, 93].

We conducted functional analyses to understand whether changes in bird assemblages are likely to extend beyond just increases or decreases in species richness. As mentioned earlier, because of their disproportionate influence on overall results, we excluded the eight Arctic region parks and Sable Island National Park Reserve from the functional analyses. In summer, only three parks (i.e., Point Pelee, Mount Revelstoke and Gulf Islands) are projected to experience a decrease in species richness, functional richness, and functional dispersion. Such a decrease in all three attributes suggests that the future assemblage may exhibit less adaptive capacity and resilience to disturbance and environmental conditions [94]. There are 22 parks, mainly across the Boreal, Atlantic Maritime, and Mixedwood Plain regions, that are projected to experience decreases in species richness and functional richness, but increases in functional dispersion. This suggests that while there may be a loss of extreme or specialized species (which can inflate the area that defines functional richness in trait space), that the predominant species in the future assemblage would be more spread out in a reduced overall space, thus may maintain a greater degree of diversity to respond to specific changes [27, 31, 94]. A decrease in functional dispersion, on the other hand, would suggest a shift towards the trait centroid for that park, with an assemblage of species with more similar traits.

In winter, most parks are projected to experience increases in both species richness and functional richness, suggesting that new species colonising the parks will increase trait combinations (bring novel trait combinations). The greatest differences in winter are with respect to functional dispersion, with many parks in the Boreal and Atlantic Maritime regions increasing in species richness, functional richness, and functional dispersion, whereas many parks in the Mixedwood Plain, Pacific Maritime, and Taiga regions are increasing in species richness and functional richness, but decreasing in functional dispersion. The former scenario, where all three attributes are projected to increase, suggests an assemblage that may have more adaptive capacity and resilience to an uncertain future [95]. The latter suggests that while some species are bringing in new trait combinations (inflating the trait space), many of the colonising species may be similar in functional traits (closer to the trait centroid). In general, the analyses suggest varied changes in the functional traits across assemblages, and as with other studies [33, 96], we recommend further examination of how these projected changes in species composition and functional traits may affect ecosystem structure and function.

While uncertainty exists around the precise rate and impacts of climate change, it is clear that conservation practices that assume stationarity, i.e., “the idea that natural systems fluctuate within an unchanging envelope of variability” [97], are no longer valid [98, 99] and that forward-looking, adaptive strategies are needed [10, 100–102]. To protect species and ecosystems, conservation practices have traditionally focused on resisting change or building resilience to support post-disturbance recovery to a historic state (i.e., persistence, [103]). However, as climate change continues to accelerate, this may no longer be tenable [104, 105] and efforts to maintain or restore the “composition and abundance of native species” and “supporting processes” (e.g., as per the definition of ecological integrity in Canada National Parks Act, [36]) may no longer be practical or possible in every park [10, 106]. As species are lost or gained in parks and ecosystems transform into potentially novel states, management will be faced with either accepting the outcome or attempting to direct it in part or in whole [98, 107, 108]. Managing for change would not only be a departure from traditional approaches, but also presents social and ecological challenges regarding difficult and unavoidable decisions about future desired states and conservation goals [32, 101, 109]. Park-level responses may need to consider not only changes to the status of individual species, but also the functional traits associated with them.

To help managers identify priorities, Fig 2 identifies each park’s anticipated trend group based on projected changes in species distribution. The parks expected to have low or intermediate change in species composition are most suitable to be managed for persistence. In these, landscape-scale bird conservation can be pursued by emphasizing actions that protect species in situ and help to build or maintain system resilience, including managing disturbances within the historic range of variability, practicing climate-smart restoration, and reducing exposure to non-climatic stresses (e.g., invasive species, fragmentation, road mortality, pollutants) [107, 110]. However, for parks in the high turnover, high potential colonisation, and high potential extirpation trend groups, managing for change is likely to be more effective with a focus on maintaining ecological structure and function, diversity, and redundancy of functional traits [111, 112]. Of particular concern are parks that also face a high loss in functional richness (e.g., Waterton Lakes, Rouge, Prince Albert, Riding Mountain, Bruce Peninsula, Cape Breton Highlands). This loss could be mitigated by facilitating the ability of species to adapt, such as improving habitat connectivity at a landscape scale, more intensive management actions (e.g., protection of key structures and functions such as nest sites), accepted or directed transformations (e.g., adaptive management, scenario-based planning, recalibration of management goals for novel ecosystems), and providing support for public engagement, social learning and good governance across scales [10, 107, 110, 113]. In practice, managers may simultaneously apply aspects of managing for persistence and change, and consider new approaches to conservation in the face of climate change.

## Conclusion

Canada’s national parks, national marine conservation areas, and national urban park are vulnerable to the impacts of a rapidly changing climate [114]. By examining species distribution models for 434 bird species, we anticipate that average species turnover at parks is expected to be approximately 25% in summer and 30% in winter by mid-century. In general, we found greater potential colonisation in winter, as warmer conditions permit more species to maintain year-round residency, contrasting with an increase in potential extirpation across parks in summer. Although it is impossible to account for the full array of changes in bird assemblages, as that will be confounded by interactions with other species and habitat factors [7], it is projected that all parks in the system will experience changes in species richness and functional traits by mid-century if climate continues to track RCP8.5.

Our results highlight potentially unavoidable and difficult challenges that require adaptive and strategic responses, where conservation decisions will include managing for persistence and change [98, 107, 113, 115]. By providing projections of future species richness, functional traits, and colonisation and extirpation potential, as well as suggesting management actions, our approach provides information that may help managers prepare for and reduce their park’s vulnerabilities.

Regardless of projected changes to a particular park, it is important to recognize that protected areas and protected area networks will remain an important cornerstone and nature-based solution for conservation during this period of unprecedented environmental change [116–118]. As one final reminder, these are projections not predictions and by reducing greenhouse gas emissions and supporting efforts to sequester and store carbon, including in natural systems, we may be able to mitigate or avoid some of the worst impacts to our protected areas [39, 119].

## Supporting information

S1 Fig Park locator map. (XLSX)

S1 Table Projected future species trend for all parks, including species removed from analyses. (XLSX)

S2 Table Park-specific trend group and turnover projection. (XLSX)

S3 Table Current and projected future functional indices and species richness for all parks. (XLSX)

S4 Table Species functional traits and calculated taxon restrictedness. (XLSX)
